# Supplementary material for: Single-atom tailored atomically-precise nanoclusters for enhanced electrochemical reduction of CO2-to-CO activity
Source: Nat Commun. 2024 Feb 28;15:1843. doi: 10.1038/s41467-024-46098-x (PMC10901820; doi:10.1038/s41467-024-46098-x)
Supplement: Supplementary file 1 — Supplementary Information [file 41467_2024_46098_MOESM1_ESM.pdf]

Supplementary Information

Single-Atom Tailored Atomically-Precise Nanoclusters for Enhanced Electrochemical Reduction of CO_2-to-CO Activity

1. Henan Key Laboratory of Crystalline Molecular Functional Materials, College of Chemistry, Zhengzhou University, Zhengzhou 450001, China.
2. College of Chemistry and Pharmaceutical Engineering, Nanyang Normal University, Nanyang 473061, People's Republic of China.

Yi-Man Wang[1], Fang-Qin Yan[1], Qian-You Wang[1], Chen-Xia Du[1], Li-Ya Wang[2], Bo Li[2], Shan Wang*[1] & Shuang-Quan Zang*[1]

E-mail: zangsqzg@zzu.edu.cn; shanwang@zzu.edu.cn

DOI: XXXXX

Characterization: Single-crystal X-ray diffraction (SCXRD) was performed on a Rigaku XtaLAB Pro diffractometer using Cu Kα radiation (λ = 1.54184 Å). Powder X-ray diffraction (PXRD) were collected on a Riguku D / Max-2500PC X-ray diffractometer with Cu sealed tub (λ = 1.54178 Å). Morphology of all samples were carried out using Zeiss Sigma 500 on a scanning electron microscopy (SEM) measurement. The Poly-(Au_8-DCP@M), Poly-(Au_8-DCP), Poly-Au_8 and Poly-DCP@Fe were prepared by employing the CH660E B14145 electrochemical workstation. Fourier transform infrared (FT-IR) spectra were recorded on a Bruker ALPHA II FT-IR spectrometer. ^{1}H nuclear magnetic resonance (NMR) spectra were recorded on a Bruker DRX spectrometer operating at 400 MHz. Transmission electron microscopy (TEM) images were obtained in FEI TalosF200S. Aberration-corrected HAADF-STEM (AC HAADF-STEM) images were obtained in FEI Titan cubed Themis G2 300 STEM with aspherical aberration corrector. The produced gas was monitored by Agilent GC7820 Gas Chromatograph (N_2 as gas carrier, and the columns of GC are Porapak Q and MolSieve 5A). The CO_2RR reaction pathways of as-prepared catalysts were detected via in situ ATR-FTIR spectrometer (BRUKER INVENIO S).

Computational Calculation. All the calculations were performed within the framework of the density functional theory (DFT) as implemented in the Vienna Ab initio Software Package (VASP 5.4.4) code within the Perdew–Burke–Ernzerhof (PBE) generalized gradient approximation and the projected augmented wave (PAW) method.[1-4] The cutoff energy for the plane-wave basis set was set to 450 eV. The Brillouin zone of the surface unit cell was sampled by Monkhorst–Pack (MP) grids, with k-point mesh density of $2\pi \times 0.04$ $Å^{-1}$ for structures optimizations. The convergence criterion for the electronic self-consistent iteration and force was set to 10^{-5} eV and 0.01 eV/Å, respectively. The vacuum layer of 15 Å was introduced to avoid interactions between periodic images.

The free energies of adsorbates at temperature T were estimated according to the harmonic approximation, and the entropy is evaluated using the following equation:

$$S(T) = K_B + \sum_{i}^{\text{harm DOF}} \left(\frac{\varepsilon_i}{K_B T (e^{\frac{\varepsilon_i}{K_B T}} - 1)} - \ln(1 - e^{-\frac{\varepsilon_i}{K_B T}}) \right)$$

where KB is Boltzmann's constant and DOF is the number of harmonic energies (εi) used in the summation denoted as the degree of freedom, which is generally 3N, where N is the number of atoms in the adsorbates. Meanwhile, the free energies of gas phase species are corrected as:

$$G_g(T) = E_{elec} + E_{ZPE} + \int C_p \, dT - TS(T)$$

where Cp is the gas phase heat capacity as a function of temperature derived from Shomate equations and the corresponding parameters in the equations were obtained from NIST.

Supplementary Table 1. Crystal data and structure refinements.

Complex	Au_8
CCDC	2290525
Empirical formula	$C_{148}H_{128}Au_8N_2P_8$
Formula weight	3758.05
Temperature/K	200(10)
Crystal system	monoclinic
Space group	*C*2/c
a (Å)	40.48288(17)
b (Å)	17.18787(7)
c (Å)	24.82685(10)
α (°)	90
β (°)	103.6391(4)
γ (°)	90
Volume ($Å^3$)	16787.73(12)
Z	4
ρ/Mg cm^{-3}	1.492
μ/mm^{-1}	13.858
F(000)	7153.0
Crystal size/mm	0.03 × 0.02 × 0.02
Radiation	CuKα (λ = 1.54184)
2Θ range for data collection/°	5.612 to 147.518
Index ranges	-49 ≤ h ≤ 48, -18 ≤ k ≤ 21, -28 ≤ l ≤ 30
Reflections collected	54141
Independent reflections	16597 [R_{int} = 0.0329, R_{sigma} = 0.0324]
Data/restraints/parameters	16597/0/748
GOF on F^2	1.069
Final R indexes [I > = 2σ (I)]	R_1 = 0.0239, wR_2 = 0.0612
Final R indexes [all data]	R_1 = 0.0270, wR_2 = 0.0624
Largest diff. peak/hole/e $Å^{-3}$	0.81 / -0.73

$R_1 = \sum||F_o|-|F_c|/\sum|F_o|$. $wR_2 = [\sum w(F_o^2 - F_c^2)^2/\sum w(F_o^2)^2]^{1/2}$

Supplementary Table 2. Bond lengths for Au_8cpz.

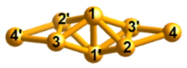

Au-Au bond	Bond length (Å)
Au1-Au1'	2.6178(2)
Au1-Au2	2.84212(18)
Au1-Au2'	2.85219(18)
Au1-Au3'	2.90083(18)
Au1-Au3	2.81354(19)
Au2-Au1'	2.85216(18)
Au2-Au3'	2.64965(19)
Au2-Au4	3.0677(2)
Au3-Au1'	2.90082(18)
Au3-Au2'	2.64966(19)
Au3-Au4'	3.1028(2)
Au4-Au3'	3.1027(2)

Supplementary Table 3. Bond angles for Au_8cpz.

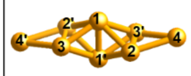

Atom-Atom-Atom	Angle/°
Au1'-Au1-Au2	62.827(6)
Au1'-Au1-Au2'	62.437(5)
Au1'-Au1-Au3'	61.048(6)
Au1'-Au1-Au3	64.447(6)
Au2-Au1-Au2'	125.264(5)
Au2-Au1-Au3'	54.940(5)
Au2'-Au1-Au3'	97.363(5)
Au3-Au1-Au2'	55.761(5)
Au3-Au1-Au2	99.632(6)
Au3-Au1-Au3'	125.495(5)
Au1-Au2-Au1'	54.737(5)
Au1'-Au2-Au4	123.612(6)
Au1-Au2-Au4	113.904(6)
Au3'-Au2-Au1	63.656(5)
Au3'-Au2-Au1'	61.380(5)
Au3'-Au2-Au4	65.254(5)
Au1-Au3-Au1'	54.503(5)
Au1-Au3-Au4'	123.712(6)
Au1'-Au3-Au4'	111.214(6)
Au2'-Au3-Au1	62.858(5)
Au2'-Au3-Au1'	61.405(5)
Au2'-Au3-Au4'	63.888(5)
Au2-Au4-Au3'	50.856(4)

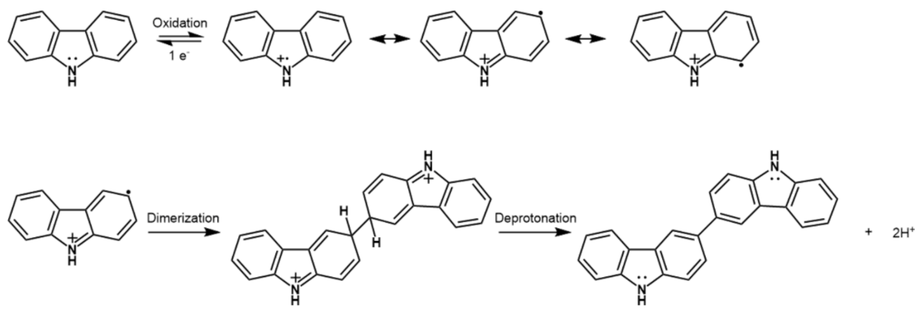

Supplementary Fig. 1 Eletropolymerization mechanism of carbazole. Mechanism of monomer oxidation, crosslinking, and reduction during the CV scans.

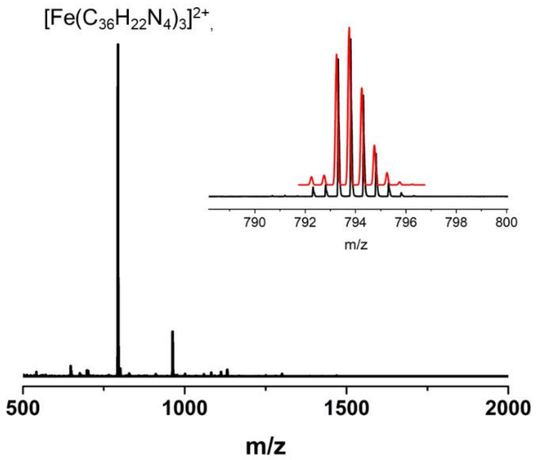

Supplementary Fig. 2 Structural characterization of DCP@Fe. Positive mode ESI-MS of the DCP@Fe ($[Fe(C_{36}H_{22}N_4)_3]^{2+}$, m/z = 793.75).

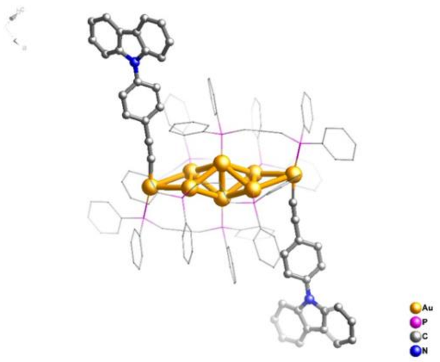

Supplementary Fig. 3 Crystal structure of Au_8 crystal. Views of crystal structure of Au_8 crystal. All H-atoms have been omitted for clarity (orange = Au, purple = P, gray = C, blue = N).

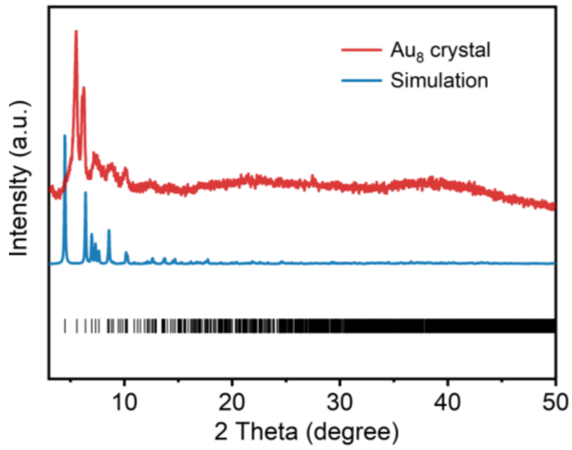

Supplementary Fig. 4 Single-crystal PXRD characterizations. PXRD patterns of simulated Au_8 and single crystal of Au_8.

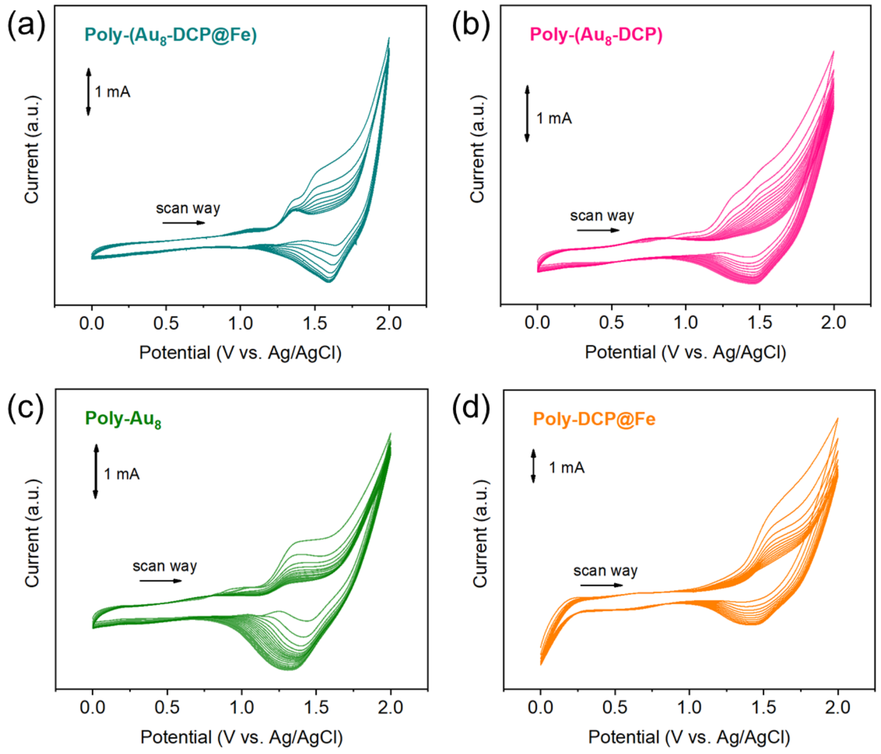

Supplementary Fig. 5 CV profiles of the electropolymerization process recorded on CP electrode. CV profiles of the electropolymerization process of (a) Au_8 and DCP@Fe, (b) Au_8 and DCP, (c) Au_8 and (d) DCP@Fe recorded on CP electrode (area = 1 × 1 cm^{-2}).

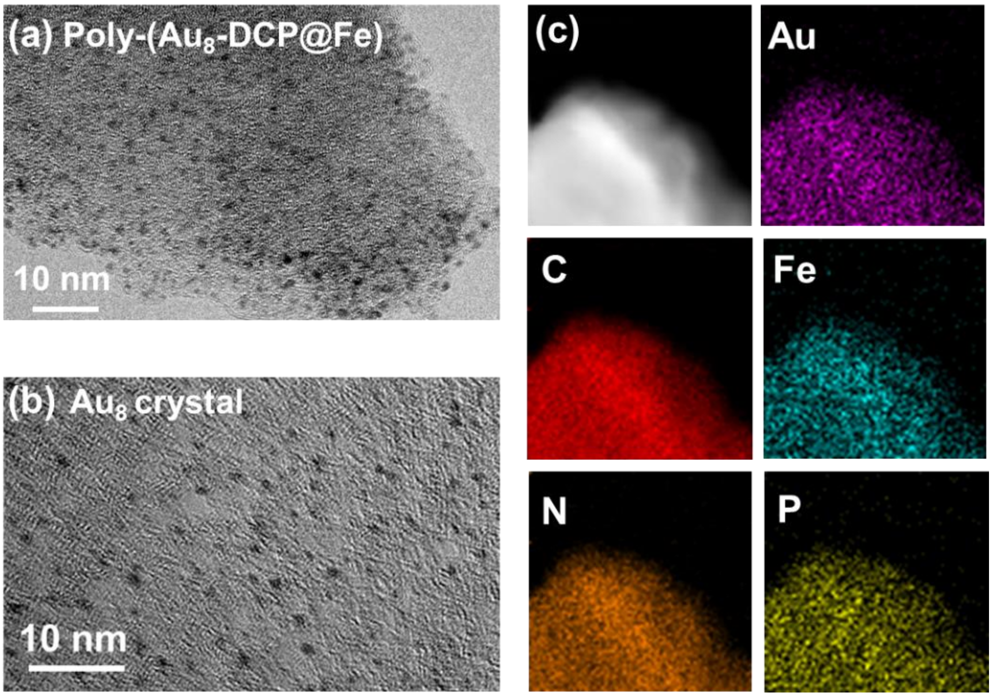

Supplementary Fig. 6 TEM and EDS characterizations of Poly-(Au_8-DCP@Fe) and Au_8 crystal. TEM images of (a) Poly-(Au_8-DCP@Fe) and (b) Au_8 crystal, (c) EDS mapping of Poly-(Au_8-DCP@Fe).

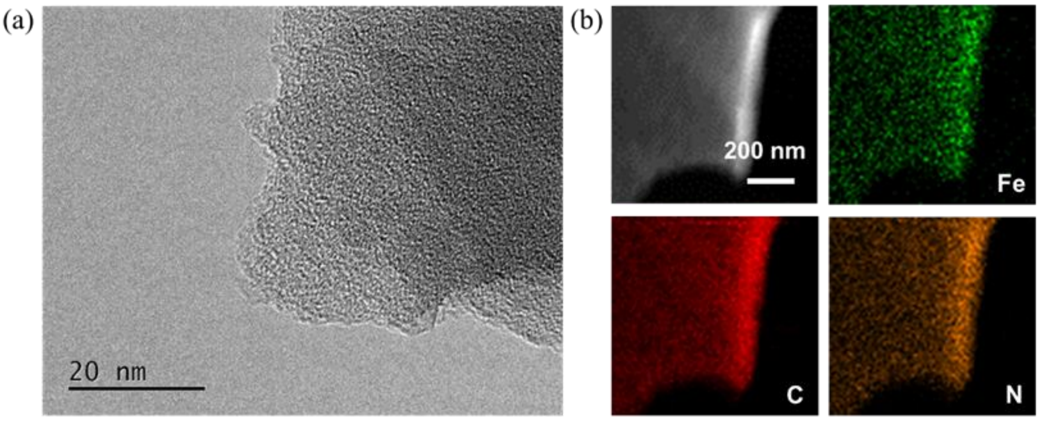

Supplementary Fig. 7 TEM and EDS characterizations of Poly-DCP@Fe. (a) TEM image and (b) Elemental mapping of Poly-DCP@Fe.

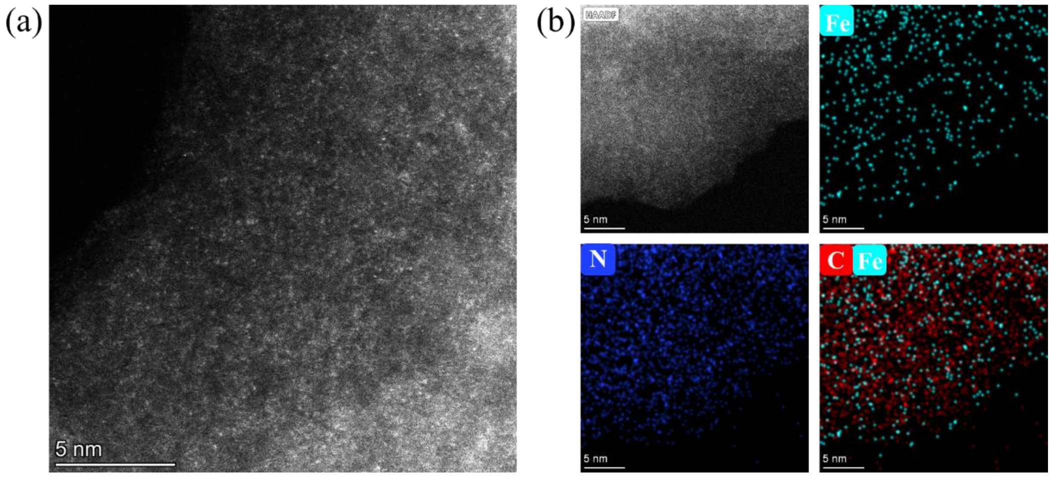

Supplementary Fig. 8 HAADF-STEM and EDS characterizations of Poly-DCP@Fe. (a) Atomic-resolution HAADF-STEM image of Poly-DCP@Fe revealing the atomically dispersed Fe SA. (b) Elemental mapping of overlapped images and C(red), Fe (cyan) and N (blue).

Supplementary Table 4 The weight percentage of Au or Fe element in Au_8 crystal, Poly-Au_8, Poly-(Au_8-DCP), Poly-(Au_8-DCP@Fe), Poly-DCP@Fe and DCP@Fe and Poly-(BCP-DCP@Fe).

	**Au%**	**Fe%**
Au_8 crystal	42.58	–
Poly-Au_8	45.24	–
Poly-(Au_8-DCP)	8.92	–
Poly-(Au_8-DCP@Fe)	5.99	4.00
Poly-DCP@Fe	–	3.56
DCP@Fe	–	4.68

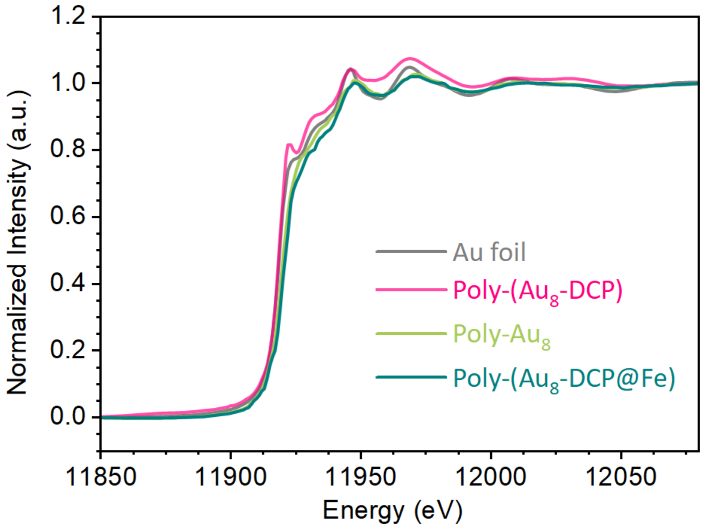

Supplementary Fig. 9 Au L3-edge XANES spectra for the catalysts. The Au L3-edge XANES of Au foil, Poly-Au8 and Poly-(Au8-DCP@Fe).

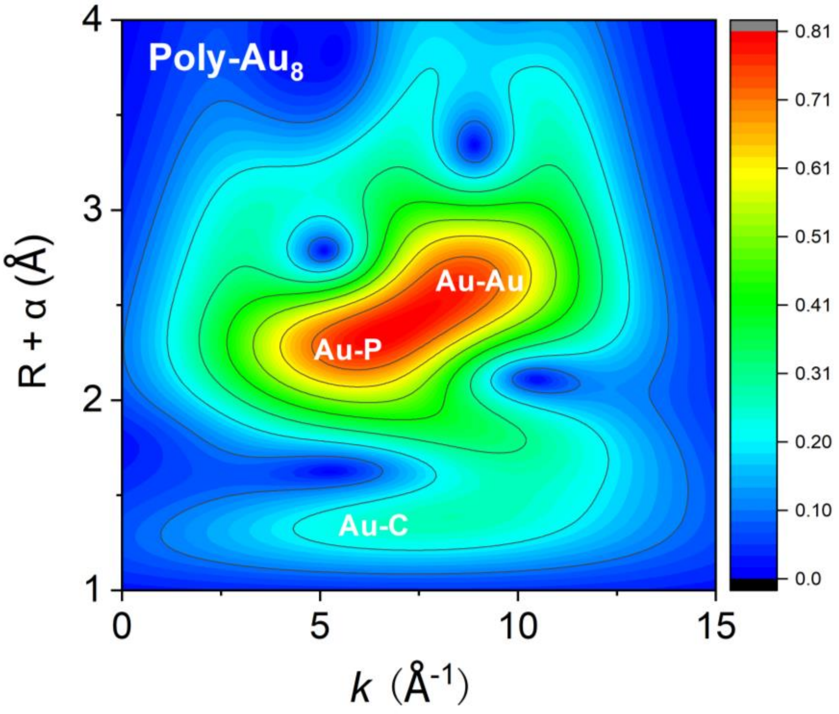

Supplementary Fig. 10 The wavelet transform spectra of Poly-Au8. Wavelet transform of Au L3-edge EXAFS for Poly-Au8.

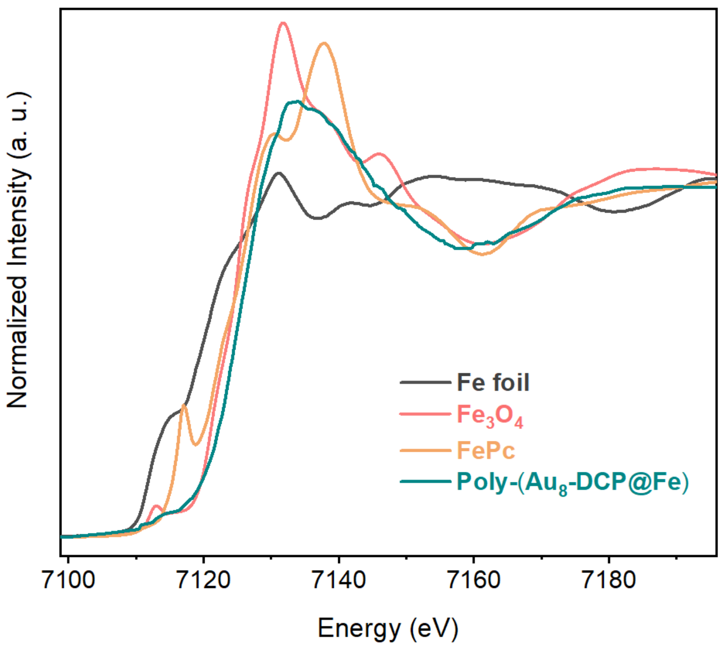

Supplementary Fig. 11 Fe *K*-edge XANES spectra for the catalysts. The Fe *K*-edge XANES of Fe foil, Fe_3O_4, FePc and Poly-(Au_8-DCP@Fe).

Supplementary Table 5. EXAFS fitting parameters at the Au L_3-edge and Fe K-edge for various samples（S_0^2=0.83）

	shell	CN	R(Å)	σ^2	ΔE_0	R factor
**Fe foil**	Fe-Fe1	8	2.47±0.01	0.0047	6.5±0.8	0.0033
	Fe-Fe2	6	2.85±0.01	0.0060		
**Au foil**	Au-Au	12	2.86±0.01	0.0081	4.8±0.3	0.0016
**Poly-(Au$_8$-DCP@Fe)**	Au-C	0.9±0.3	1.95±0.02	0.0042	-15	
	Au-P	0.7±0.3	2.41±0.04	0.0114	-15	0.0163
	Au-Au	10.2±0.8	2.86±0.01	0.0152	7.0±1.3	
	Fe-N	5.9±0.6	2.02±0.02	0.0103	0.6±3.0	0.0121

^{a}*CN*: coordination numbers; ^{b}*R*: bond distance; $^c\sigma^2$: Debye-Waller factors; ^{d} ΔE_0: the inner potential correction. *R* factor: goodness of fit.

The obtained XAFS data was processed in Athena (version 0.9.26) for background, pre-edge line and post-edge line calibrations. Then Fourier transformed fitting was carried out in Artemis (version 0.9.26). The k^2 weighting, k-range of 3 – ~11.5 Å^{-1} and R range of 1 - 3 Å were used for the fitting of Au foil and Sample. The four parameters, coordination number, bond length, Debye-Waller factor and E_0 shift (CN, R, ΔE_0) were fitted without anyone was fixed, the σ^2 was set.

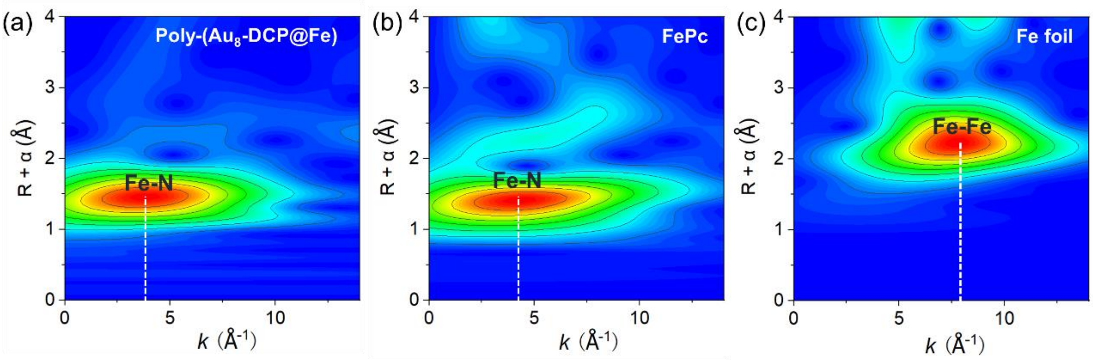

Supplementary Fig. 12 Wavelet transform of Poly-(Au$_8$-DCP@Fe) and control materials. Wavelet transform of Fe *K*-edge EXAFS for (a) Poly-(Au$_8$-DCP@Fe), (b) FePc and (c) Fe foil.

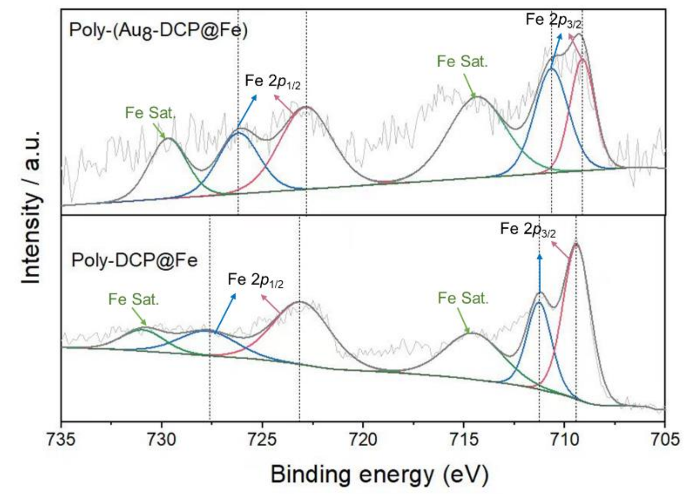

Supplementary Fig. 13 Comparison of the XPS spectras over Poly-(Au_8-DCP@Fe) and Poly-DCP@Fe electrode. XPS spectra of Fe 2*p* for Poly-(Au_8-DCP@Fe) and Poly-DCP@Fe.

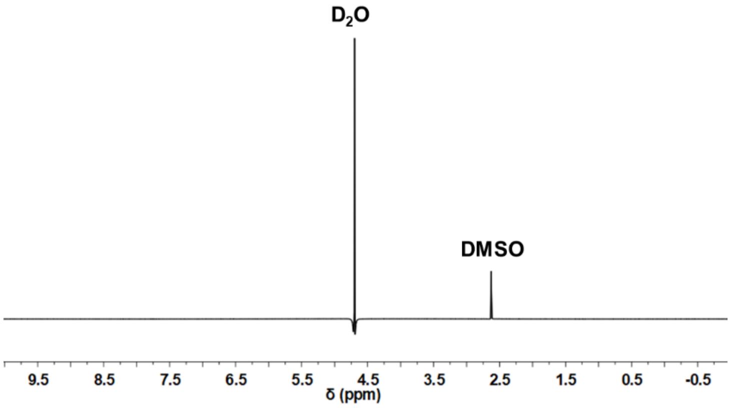

Supplementary Fig. 14 Liquid phase product analysis. ^{1}H NMR spectroscopy of the reaction mixture of Poly-(Au_8-DCP@Fe) after catalysis, DMSO was added as internal standard.

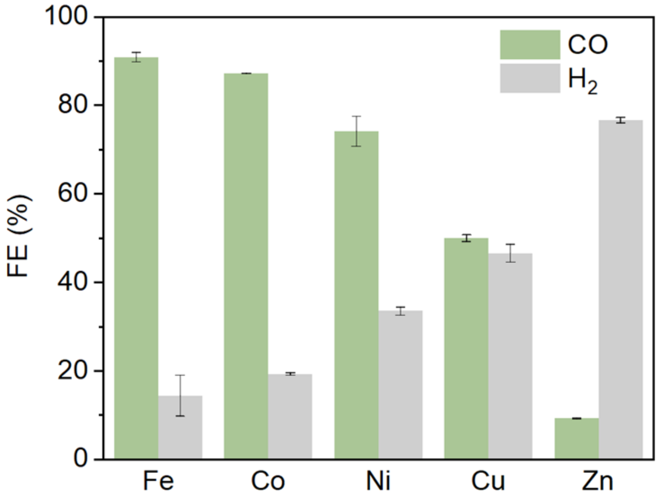

Supplementary Fig. 15 CO FE and H_2 FE of Poly-(Au_8-DCP@M). Faradaic efficiencies of Poly-(Au_8-DCP@M) at different applied potentials in CO_2-saturated 0.5 M $KHCO_3$ aqueous solution.

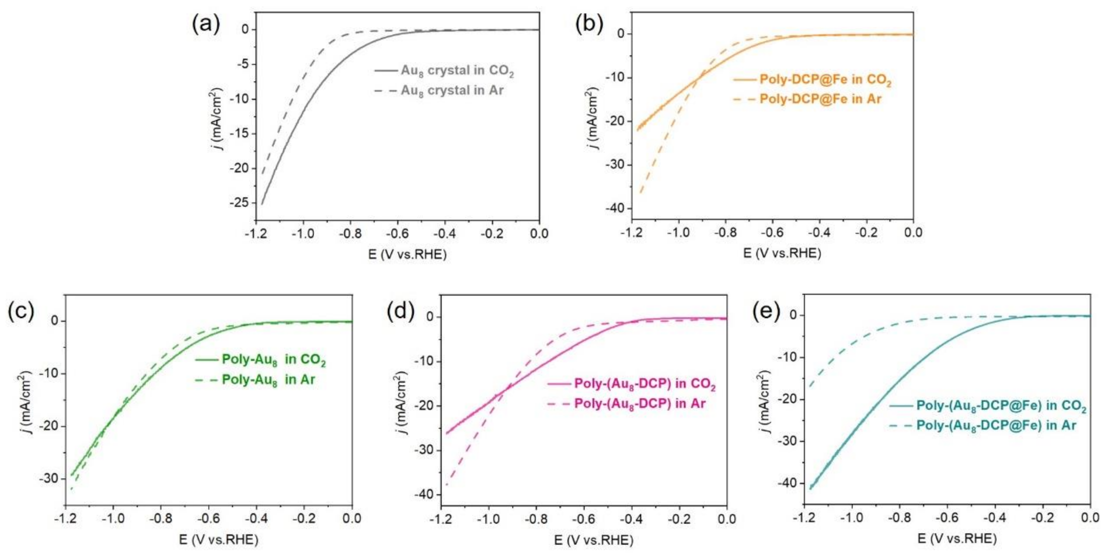

Supplementary Fig. 16 LSV curves of various catalysts in CO_2-saturated or Ar - saturated $KHCO_3$ solution. LSV curves for (a) Au_8 crystal (b) Poly-DCP@Fe (c) Poly-Au_8 (d) Poly-(Au_8-DCP) and (e) Poly-(Au_8-DCP@Fe) in a CO_2-saturated and Ar-saturated $KHCO_3$ solution at the potential range of 0 to -1.17 V (vs RHE, scan rate of 50 mV/s).

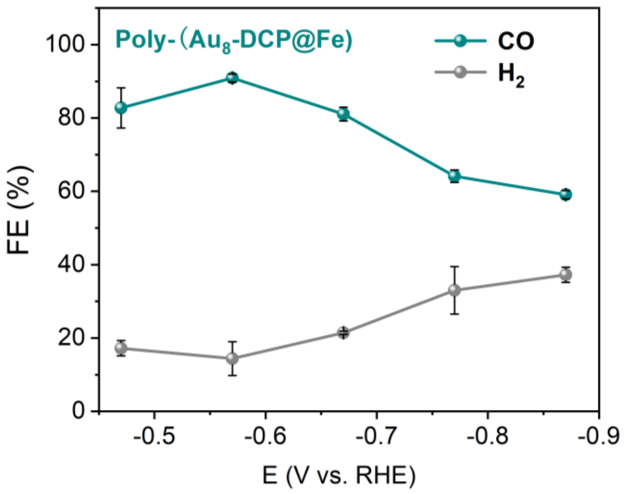

Supplementary Fig. 17 Electrocatalytic CO_2 reduction performance of Poly-(Au_8-DCP@Fe). Faradaic efficiencies of Poly-(Au_8-DCP@Fe) at different applied potentials in CO_2-saturated 0.5 M $KHCO_3$ aqueous solution.

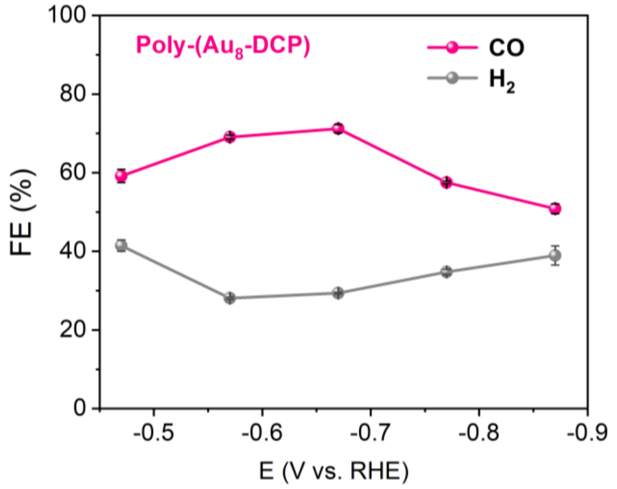

Supplementary Fig. 18 Electrocatalytic CO_2 reduction performance of Poly-(Au_8-DCP). Faradaic efficiencies of Poly-(Au_8-DCP) at different applied potentials in CO_2-saturated 0.5 M $KHCO_3$ aqueous solution.

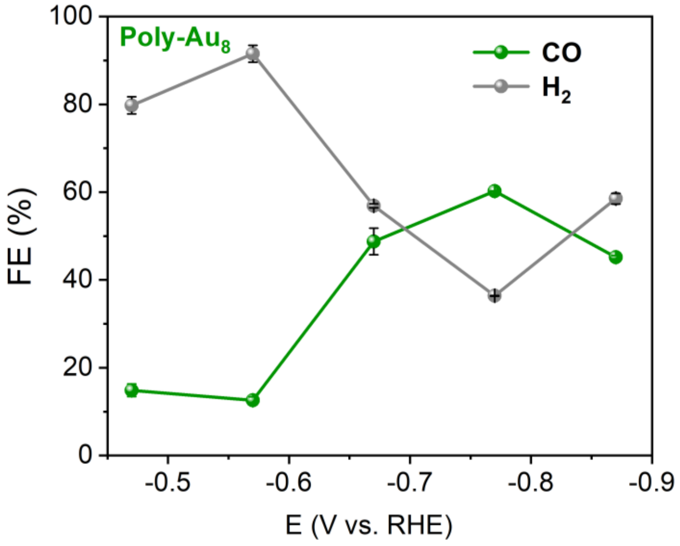

Supplementary Fig. 19 Electrocatalytic CO_2 reduction performance of Poly-Au_8. Faradaic efficiencies of Poly-Au_8 at different applied potentials in CO_2-saturated 0.5 M $KHCO_3$ aqueous solution.

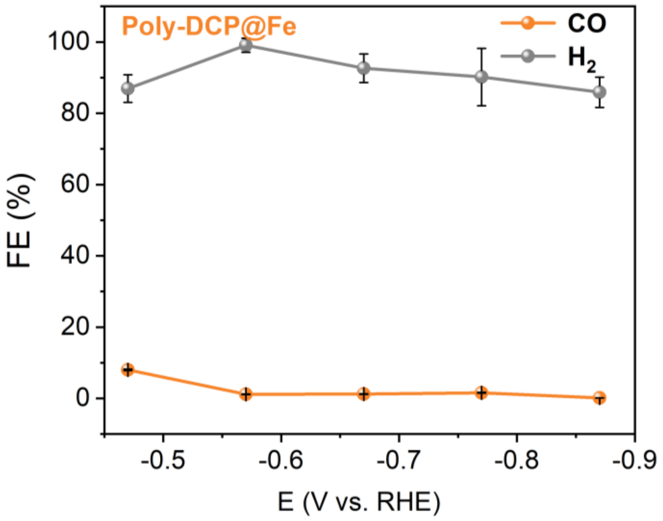

Supplementary Fig. 20 Electrocatalytic CO_2 reduction performance of Poly-DCP@Fe. Faradaic efficiencies of Poly-DCP@Fe at different applied potentials in CO_2-saturated 0.5 M $KHCO_3$ aqueous solution.

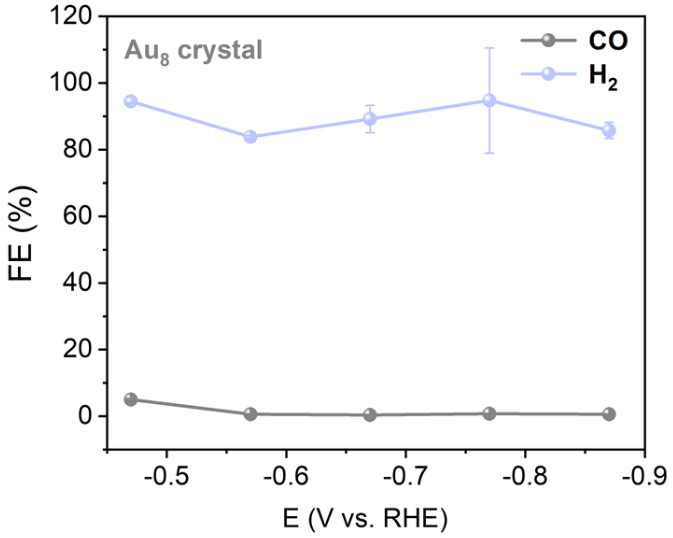

Supplementary Fig. 21 Electrocatalytic CO_2 reduction performance of Au_8 crystal. Faradaic efficiencies of Au_8 crystal at different applied potentials in CO_2-saturated 0.5 M $KHCO_3$ aqueous solution.

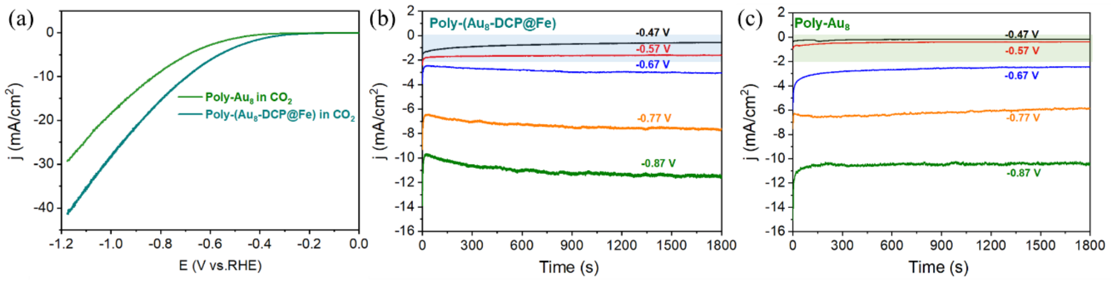

Supplementary Fig. 22 Characterization of current density for Poly-Au_8 and Poly-(Au_8-DCP@Fe). (a) LSV curves for Poly-Au_8 and Poly-(Au_8-DCP@Fe) in a CO_2-saturated $KHCO_3$ solution at the potential range of 0 to -1.17 V (vs RHE, scan rate of 50 mV/s). (b, c) Chronoamperometric responses of (b) Poly-(Au_8-DCP@Fe) and (c) Poly-Au_8 at different potentials (−0.47 to −0.87 V, vs. RHE).

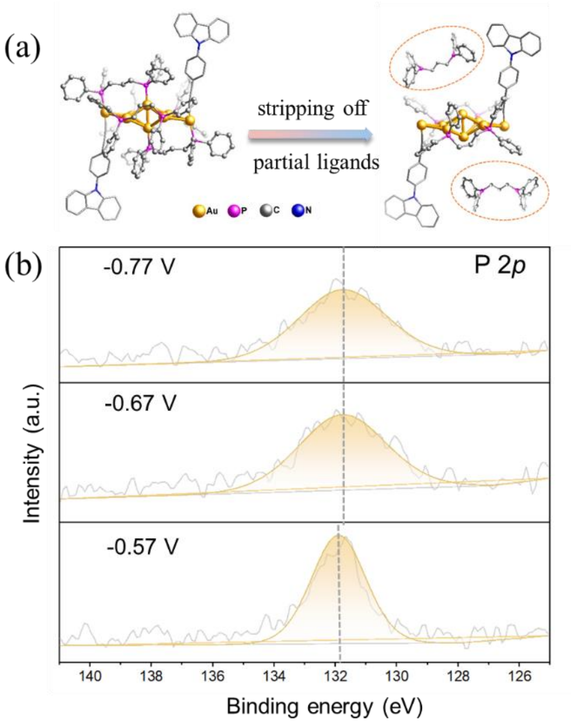

Supplementary Fig. 23 *Quasi-in situ* XPS of Poly-Au_8 during the CO_2RR process at -0.57 V, -0.67 V and -0.77 V. (a) Schematic illustration of Poly-Au_8 partial bidentate pisphosphine ligands removal at -0.67 V and -0.77 V. (b) The *Quasi-in situ* XPS spectra of P 2*p* for Poly-Au_8 at -0.57 V, -0.67 V and -0.77 V (vs. RHE).

Quasi-in situ XPS of Poly-Au_8 revealing changes during the CO_2RR process at -0.57 V, -0.67 V and -0.77 V. The broadening of P 2*p* and N 1s peaks after -0.57 V vs RHE suggests the formation of defects due to partial removal of ligands, exposing catalytic sites and enhancing CO_2RR efficiency. Additionally, the shift in P 2*p* to lower binding energies indicates an increased electron cloud density of ligands, which supports stripping off the strong electron-donated bidentate pisphosphine ligands. This result may explain the significant increase in CO FE after -0.57 V vs RHE for Poly-Au_8.

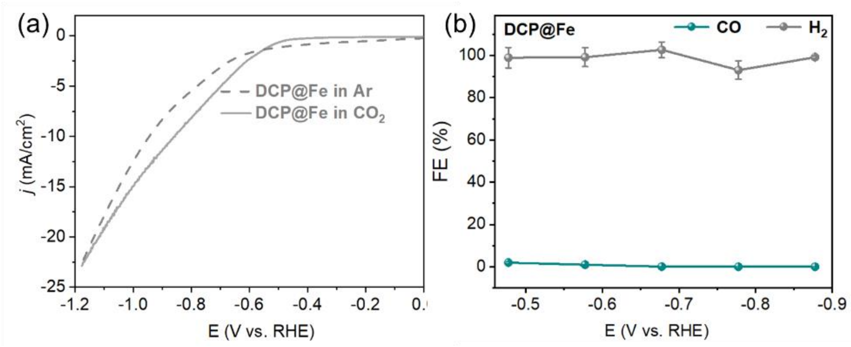

Supplementary Fig. 24 Electrocatalytic CO_2 reduction performance of DCP@Fe. (a) LSV curves for DCP@Fe in a CO_2-saturated and Ar-saturated $KHCO_3$ solution at the potential range of 0 to -1.17 V (vs RHE, scan rate of 50 mV/s). (b)Faradaic efficiencies of DCP@Fe at different applied potentials in CO_2-saturated 0.5 M $KHCO_3$ aqueous solution.

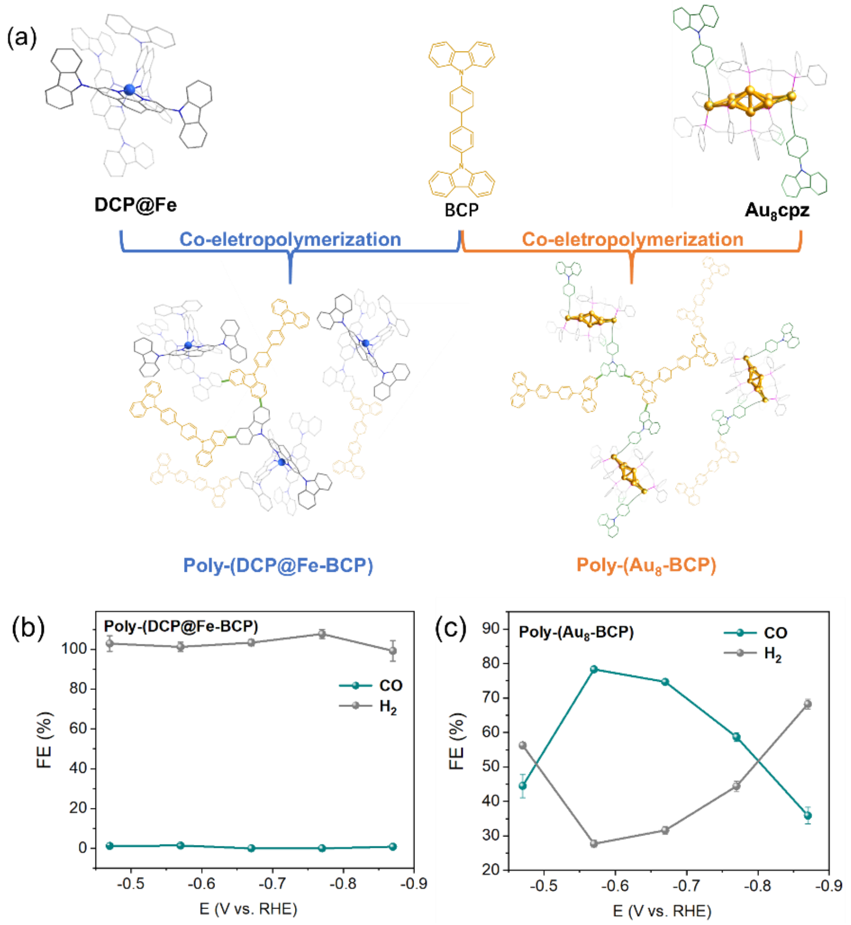

Supplementary Fig. 25 Schematic illustration and electrocatalytic CO_2 reduction performance of Poly-(DCP@Fe-BCP) and Poly-(Au_8-BCP). (a) Schematic illustration of Poly-(DCP@Fe-BCP) and Poly-(Au_8-BCP) fabricated by co-eletropolymerization strategy. Faradaic efficiencies of (b) Poly-(DCP@Fe-BCP) and (c) Poly-(Au_8-BCP) at different applied potentials in CO_2-saturated 0.5 M $KHCO_3$ aqueous solution.

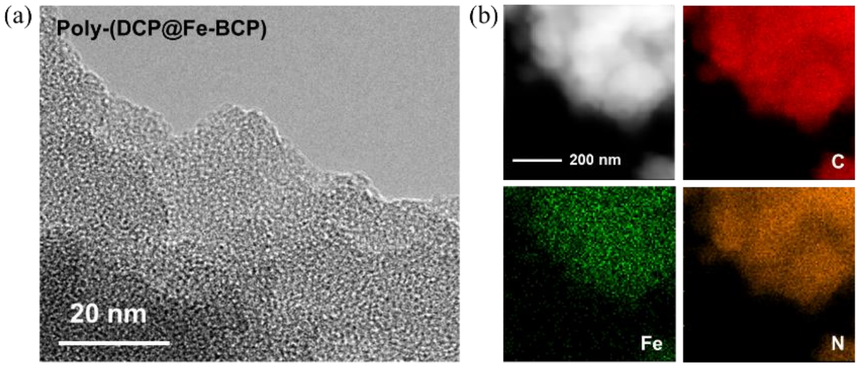

Supplementary Fig. 26 TEM and EDS characterizations. (a) TEM image and (b) Elemental mapping of Poly-(DCP@Fe-BCP).

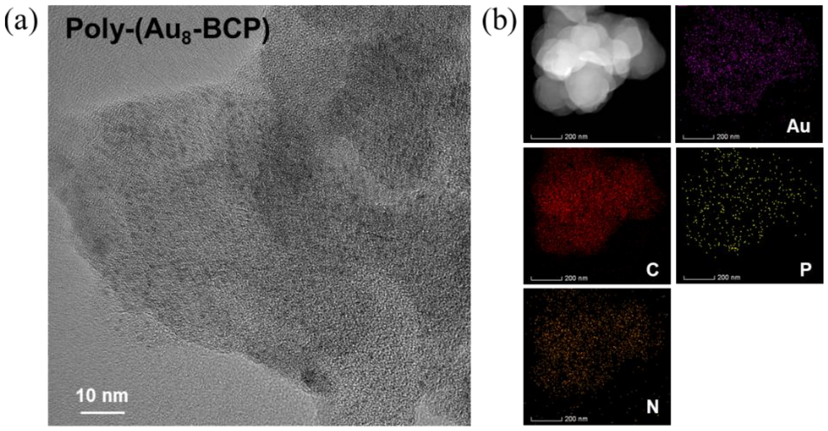

Supplementary Fig. 27 TEM and EDS characterizations. (a) TEM image and (b) Elemental mapping of Poly-(Au_8-BCP).

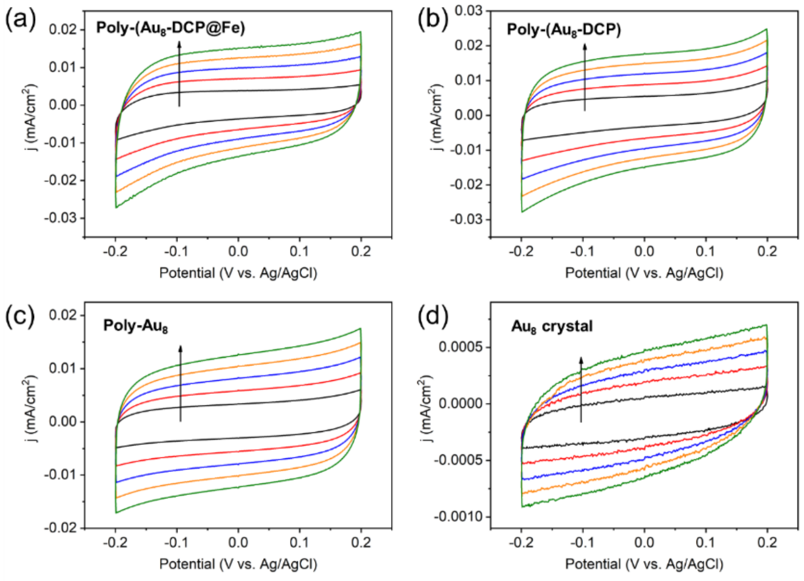

Supplementary Fig. 28 CV curves of catalysts with various scan rates. CV curves of (a) Poly-(Au_8-DCP@Fe), (b) Poly-(Au_8-DCP), (c) Poly-Au_8, and (d) Au_8 crystal with various scan rates from 10 to 50 mV s^{-1}.

Although most studies indicated a negative role of the ligands because of their blockage of the active Au sites, the presence of carbazole ligands in Au_8 NCs even showed a promotional effect on the CO_2RR catalysis in this strategy. Unlike discrete Au_8 clusters, the electropolymerized biscarbazole network enhances electron-transfer to metal catalytic sites, extending the active surface area far beyond the electrode. Electrochemical double-layer capacitance measurements confirm that Poly-Au_8 exhibits a significantly higher electrochemical surface area compared to pristine Au_8cpz

crystal, underscoring its superior performance in providing active sites for CO_2 reduction.

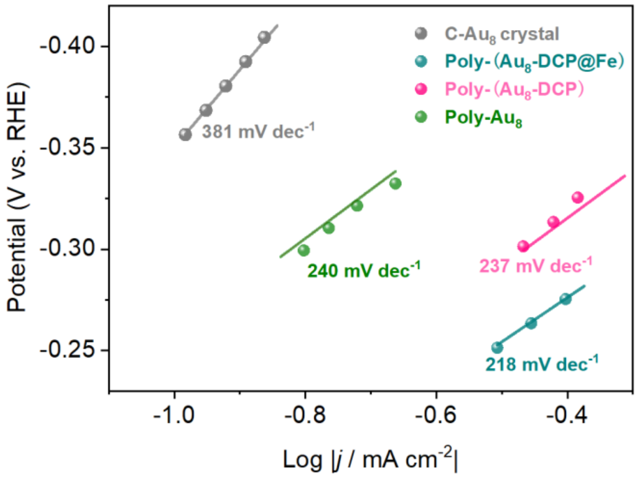

Supplementary Fig. 29 Comparison of Tafel slopes for various catalysts. Corresponding Tafel slopes for Poly-(Au_8-DCP@Fe), Poly-(Au_8-DCP), Poly-Au_8, Poly-DCP@Fe, and Au_8 crystal.

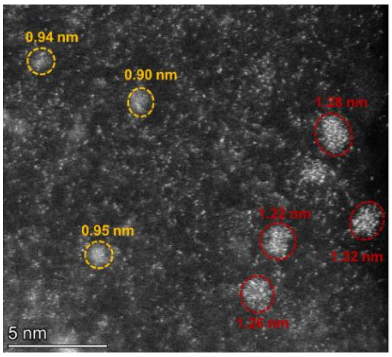

Supplementary Fig. 30 HAADF-STEM characterizations after stability test. HAADF-STEM image of Poly-(Au_8-DCP@Fe) after stability test.

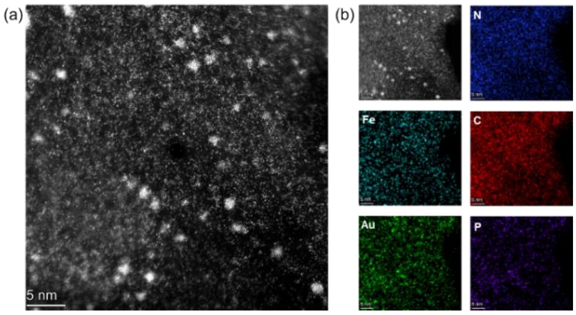

Supplementary Fig. 31 HAADF-STEM and EDS characterizations after stability test. (a) HAADF-STEM image and (b) Elemental mapping of Poly-(Au_8-DCP@Fe) after stability test.

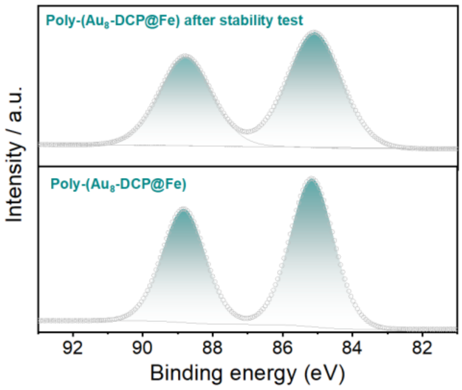

Supplementary Fig. 32 Comparison of the XPS spectras over Poly-(Au_8-DCP@Fe) electrode before and after stability test. XPS spectras of Au 4*f* in Poly-(Au_8-DCP@Fe) before and after stability test.

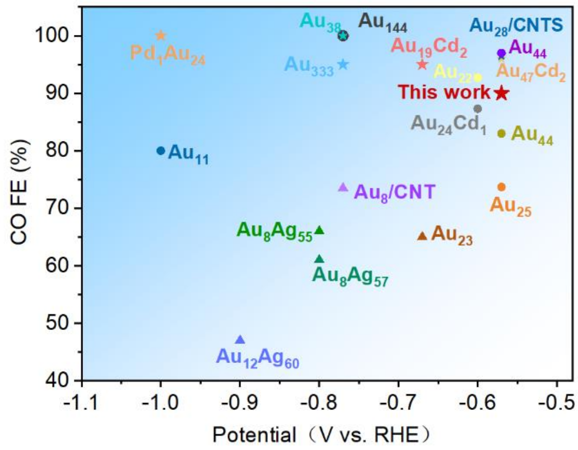

Supplementary Fig. 33 CO FE of representative Au NCs catalysts. CO FE of Poly-(Au_8-DCP@Fe) compared with other representative Au NCs catalysts.

Supplementary Table 6 Comparison of CO_2RR performance of Poly-(Au_8@DCP@Fe) with other representative Au nanoclusters catalysts.

Cathode	electrolyte	Potential（V vs. RHE）	CO FE	Ref.
**Poly-(Au_8-DCP@Fe)**	0.5 M $KHCO_3$	**-0.57**	**90%**	This work
**Au_{25} cluster**	0.5 M $KHCO_3$	**-0.57**	**73.7%**	**5**
**Au_{11}**	0.5 M $KHCO_3$	**-1.00**	**> 80%**	**6**
**Pd_1Au_{24}**	0.1 M $KHCO_3$	**-1.00**	**100%**	**7**
**$Au_{47}Cd_2$ cluster**	0.5 M $KHCO_3$	**-0.57**	**96%**	**8**
**Au_{44} cluster**	0.5 M $KHCO_3$	**-0.57**	**83%**	**8**
**$Au_{24}Cd_1(PET)_{18}$ cluster**	0.5 M $KHCO_3$	**-0.60**	**87.3%**	**9**
**Au_{23}**	0.5 M $KHCO_3$	**-0.67**	**65%**	**10**
**$Au_{19}Cd_2$**	0.5 M $KHCO_3$	**-0.67**	**95%**	**10**
**Au_{28}/CNTs**	0.5 M $KHCO_3$	**-0.57**	**96.5 %**	**11**
**Au_{38}**	3.0 M KOH	**-0.77**	**~100%**	**12**
**Au_{144}**	3.0 M KOH	**-0.77**	**~100%**	**12**
**Au_{333}**	3.0 M KOH	**-0.77**	**95%**	**12**
**Au_{22}**	0.5 M $KHCO_3$	**-0.6**	**92.7%**	**13**
**Au_{24}**	0.1 M $KHCO_3$	**--**	**> 90%**	**14**
**Au_8Ag_{55}**	0.5 M $KHCO_3$	**-0.80**	**66%**	**15**
**Au_8Ag_{57}**	0.5 M $KHCO_3$	**-0.80**	**61%**	**15**
**$Au_{12}Ag_{60}$**	0.5 M $KHCO_3$	**-0.90**	**47%**	**15**
**Au_7/CNT**	0.5 M $KHCO_3$	**-0.67**	**1.8%**	**16**
**Au_8/CNT**	0.5 M $KHCO_3$	**-0.77**	**73.5%**	**16**
**Au_{44}-P**	0.5 M $KHCO_3$	**-0.57**	**97%**	**17**

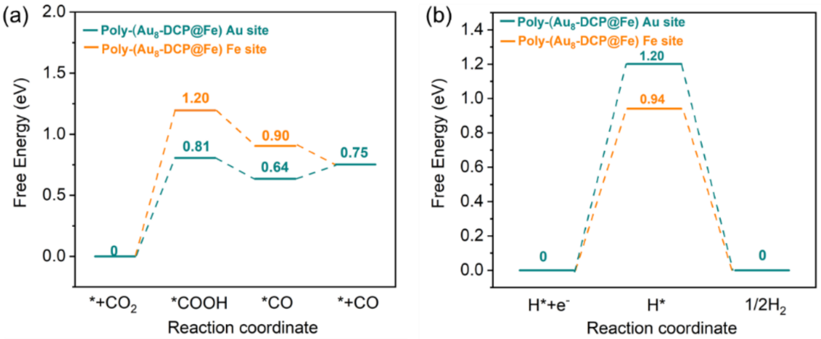

Supplementary Fig. 34 Comparison of free energy barriers at different sites of CO_2RR and HER on Poly-(Au_8-DCP@Fe). The free energy diagram of the (a) CO_2RR and (b) HER on the Fe sites and Au_8 sites of Poly-(Au_8-DCP@Fe).

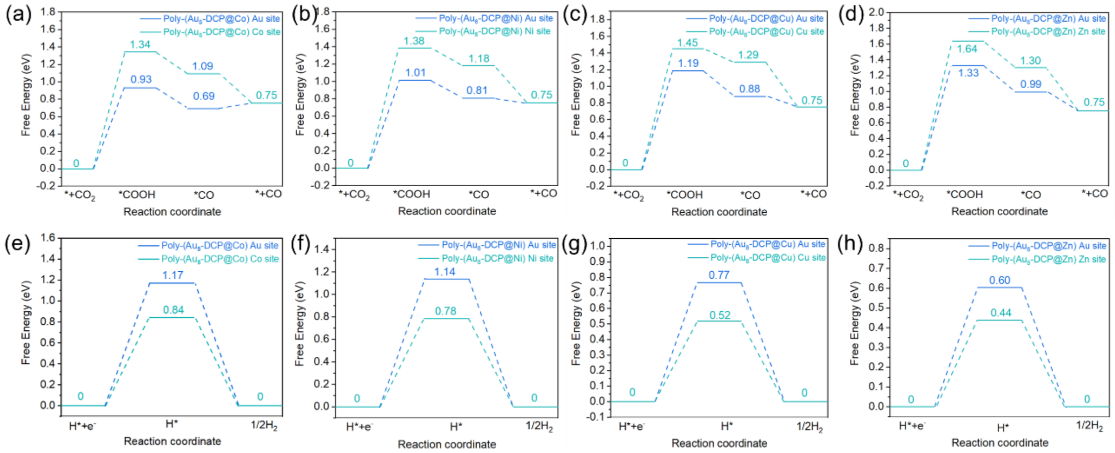

Supplementary Fig. 35 Comparison of free energy barriers between HER and CO_2RR on different metal sites. The free energy diagram of the (a-d) CO_2RR and (e-h) HER on the M sites and Au_8 sites of Poly-(Au_8-DCP@M), M = Co, Ni, Cu, Zn.

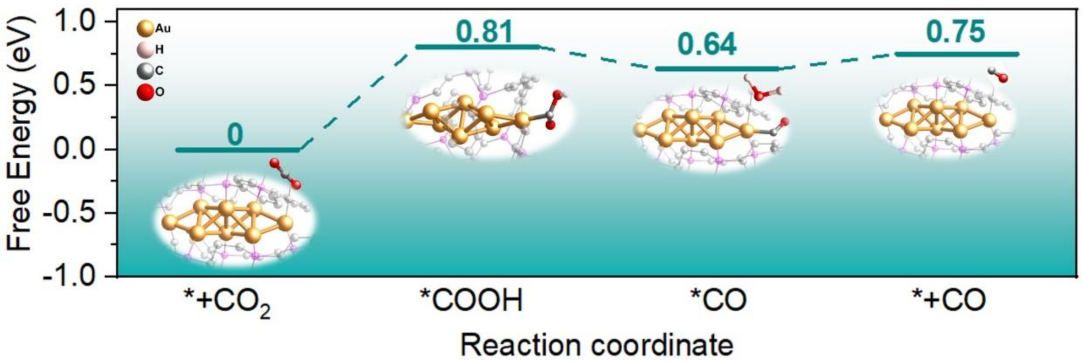

Supplementary Fig. 36 Reaction paths and free energy diagrams. Reaction paths and Free energy diagrams of CO_2 reduction to CO for Poly-(Au_8-DCP@Fe).

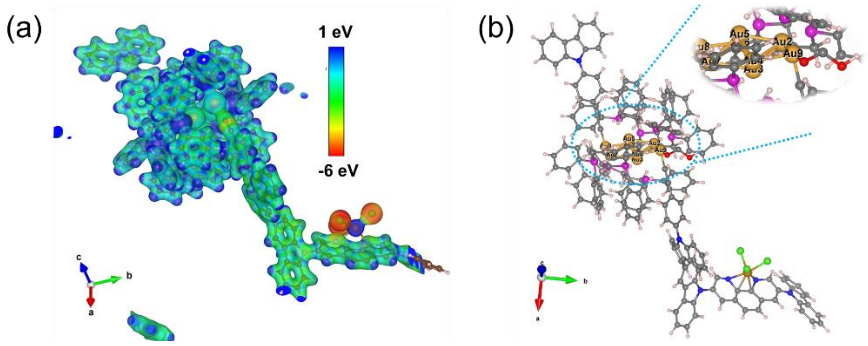

Supplementary Fig. 37 Electrostatic potential and calculated *COOH adsorption site. (a) Electrostatic potential, Vel, mapped onto the charge density isosurface for Poly-(Au_8-DCP@Fe) and (b) Calculated *COOH adsorption site on Poly-(Au_8-DCP@Fe) structure.

Supplementary References

1. Perdew, J. P.; Burke, K.; Ernzerhof, M., Generalized Gradient Approximation Made Simple. *Phys. Rev. Lett.* **77**, 3865-3868, (1996).

2. Hammer, B.; Hansen, L. B.; Nørskov, J. K., Improved adsorption energetics within density-functional theory using revised Perdew-Burke-Ernzerhof functionals. *Phys. Rev. B,* **59**, 7413-7421, (1999).

3. Blöchl, P. E., Projector augmented-wave method. *Phys. Rev. B,* **50**, 17953-17979, (1994).

4. Kresse, G.; Joubert, D., From ultrasoft pseudopotentials to the projector augmented-wave method. *Phys. Rev. B,* **59**, 1758-1775, (1999).

5 Zhao, S. et al. Influence of Atomic-Level Morphology on Catalysis: The Case of Sphere and Rod-Like Gold Nanoclusters for CO_2 Electroreduction. *ACS Catal.* **8**, 4996-5001, (2018).

6 Narouz, M. R. et al. N-heterocyclic carbene-functionalized magic-number gold nanoclusters. *Nature Chem.* **11**, 419-425, (2019).

7 Li, S. et al. Monopalladium Substitution in Gold Nanoclusters Enhances CO_2 Electroreduction Activity and Selectivity. *ACS Catal.* **10**, 12011-12016, (2020).

8 Zhuang, S. et al. Hard-Sphere Random Close-Packed $Au_{47}Cd_2(TBBT)_{31}$ Nanoclusters with a Faradaic Efficiency of Up to 96 % for Electrocatalytic CO_2 Reduction to CO. *Angew. Chem. Int. Ed.* **59**, 3073-3077, (2020).

9 Sun, Y., Liu, X., Xiao, K., Zhu, Y. & Chen, M. Active-Site Tailoring of Gold Cluster Catalysts for Electrochemical CO_2 Reduction. *ACS Catal.* **11**, 11551-11560, (2021).

10 Li, S. et al. Boosting CO_2 Electrochemical Reduction with Atomically Precise Surface Modification on Gold Nanoclusters. *Angew. Chem. Int. Ed.* **60**, 6351-6356, (2021).

11 Yuan, S. F. et al. Robust Gold Nanocluster Protected with Amidinates for Electrocatalytic CO_2 Reduction. *Angew. Chem. Int. Ed.* **60**, 14345-14349, (2021).

12 Li, S. et al. Dissecting Critical Factors for Electrochemical CO_2 Reduction on Atomically Precise Au Nanoclusters. *Angew. Chem. Int. Ed.* **61**, e202211771, (2022).

13 Gao, Z. H. et al. A Heteroleptic Gold Hydride Nanocluster for Efficient and Selective Electrocatalytic Reduction of CO_2 to CO. *J. Am. Chem. Soc.* **144**, 5258–5262 (2022).

14 Kulkarni, V. K. et al. N-Heterocyclic Carbene-Stabilized Hydrido Au_{24} Nanoclusters: Synthesis, Structure, and Electrocatalytic Reduction of CO_2. *J. Am. Chem. Soc.* **144**, 9000–9006 (2022).

15 Hu, J. et al. Evolution of Electrocatalytic CO_2 Reduction Activity Induced by Charge Segregation in Atomically Precise AuAg Nanoclusters Based on Icosahedral M(13) Unit 3D Assembly. *Small*, e2301357, (2023).

16 Tang, L. et al. Poly-Hydride $[Au^{I}_7(PPh_3)_7H_5](SbF_6)_2$ cluster complex: Structure, Transformation, and Electrocatalytic CO_2 Reduction Properties. *Angew. Chem. Int. Ed.* **62**, e202300553, (2023).

17 Zhuang, S. et al. Phosphine-Triggered Structural Defects in Au(44) Homologues Boost Electrocatalytic CO_2 Reduction. *Angew. Chem. Int. Ed.*, e202306696, (2023).
